# Nano-embossing technology on ferroelectric thin film Pb(Zr_0.3_,Ti_0.7_)O_3 _for multi-bit storage application

**DOI:** 10.1186/1556-276X-6-474

**Published:** 2011-07-27

**Authors:** Zhenkui Shen, Zhihui Chen, Qian Lu, Zhijun Qiu, Anquan Jiang, Xinping Qu, Yifang Chen, Ran Liu

**Affiliations:** 1 ASIC & System State Key Laboratory, Department of Microelectronics, Fudan University, Shanghai, 200433, China; 2 Department of Material Science, Fudan University, Shanghai, 200433, China; 3 Rutherford Appleton Laboratory, Chilton, Didcot, Oxfordshire, OX11 0QX, UK

## Abstract

In this work, we apply nano-embossing technique to form a stagger structure in ferroelectric lead zirconate titanate [Pb(Zr_0.3_, Ti_0.7_)O_3 _(PZT)] films and investigate the ferroelectric and electrical characterizations of the embossed and un-embossed regions, respectively, of the same films by using piezoresponse force microscopy (PFM) and Radiant Technologies Precision Material Analyzer. Attributed to the different layer thickness of the patterned ferroelectric thin film, two distinctive coercive voltages have been obtained, thereby, allowing for a single ferroelectric memory cell to contain more than one bit of data.

## Introduction

The development of miniaturized ferroelectric field effect transistors (FeFETs) and random access memories (FeRAMs) [1,2] has called for fabrication of high-quality ferroelectric nanostructures. How to retain excellent ferroelectricity in nanoscale patterned structures posts a great challenge, as the small thickness of ultra thin films as well as the damages and defects introduced by the conventional photo lithography [3-6] could drastically degrade the ferroelectric properties. Better controlling the quality of the ultra-thin ferroelectric films and alternative patterning techniques are, therefore, highly required.

It is widely understood that multi-bit operation could be one of the most efficient approaches to increase storage densities. In recent years, a great deal of efforts has been made on realizing multi-value storage through circuit design. One of the drawbacks is the additional budget of densities in circuit integration. There have been rarely reports on the research tackling the improvement of fabrication processes and device structures. Nano-embossing technology has emerged as a fast and cost effective technique suitable for patterning structures with feature size down to 20 nm, well below the limit of other lithography techniques used for mass production [7-11]. In this article, we report our initial progress in developing a nano-embossing technique to achieve large arrays of ferroelectric PZT cells, which have potential application in multi-bit storage based on ferroelectric nanostructures.

Since the principle of FeRAM is based on the polarization reversal by an externally applied electric field of metal-ferroelectric-metal capacitors, the computational '0'and '1' are represented by the nonvolatile storage of the negative or positive remnant polarization state, respectively [12]. The ferroelectric films with different thickness need different coercive voltages. A staggered structure with two distinct thickness layers on a PZT thin film can be readily created by a one-step embossing process. In principle, the thinner layer should give rise to the lower switch voltage and the thicker layer to the higher switch voltage [13]. The voltage magnitude corresponds to one of the two polarization charge levels stored in the ferroelectric memory cell. In this way, multiple bits of data can be obtained from a single ferroelectric memory cell by applying different voltages.

Scanning atomic force microscopy (AFM) was used to study the morphology of the embossed arrays, whereas the PFM, which is proved to be one of the most effective method for the nanoscale study and control of ferroelectric domains in bulk crystals and thin films [14,15], was applied for the study of polarization switching behavior of patterned regions on a PZT film. A Radiant Technologies Precision Material Analyzer was also used for electrical characterizations. The same measurements were also performed on un-patterned regions for comparison.

## Results and discussion

**Figure 1:**
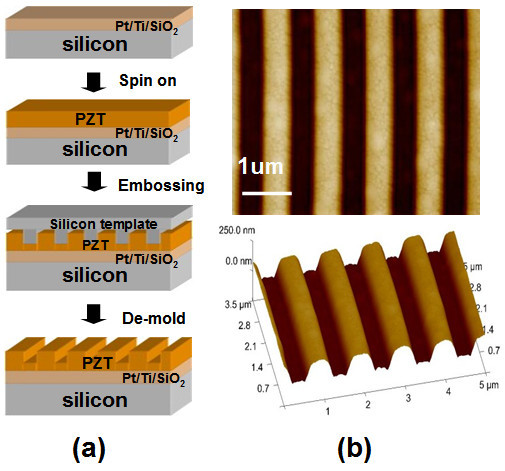
**PZT films nano-embossing process and results of embossed profiles**. **(a) **A schematic diagram illustrating a one-step embossing process to form a stagger shape in a ferroelectric PZT film with two different thicknesses. **(b) **AFM images of embossed stagger like profiles of ferroelectric PZT film.

**Figure 2:**
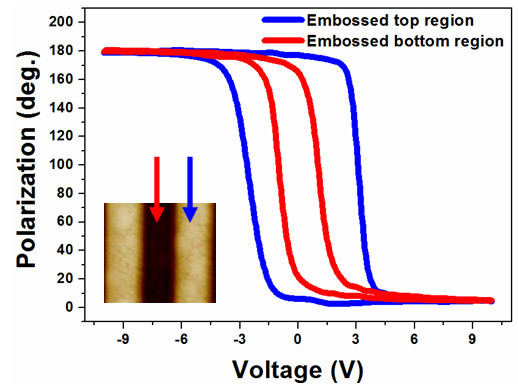
**Hysteretic dependence of OPP phase with applied voltage from -10 to 10 V for embossed top (blue) and bottom regions (red)**.

Figure 1a illustrates the nano-embossing process of ferroelectric PZT. Figure 1b displays the embossed PZT film profiles measured by AFM; showing an embossed depth of about 160 nm on a 450-nm thick PZT film. It could be seen that well quadrate patterned profiles of the PZT film has been obtained by the nano-embossing process. After crystallization, the morphology of the embossed region remained stable and no collapse was found even after several months. The embossed PZT films were found to be with [111] preferred orientation and a tetragonal structure previously by X-ray diffraction and Raman spectroscopy (not presented here) [13,16], respectively (Additional files 1 and 2). Figure 2 shows the hysteretic dependence of out-of-plane piezoresponse (OPP) phase under a bias from -10 to 10 V. The blue and red curves represent the hysteresis loops of embossed top and bottom areas, respectively. The polarization phase contrast is nearly 180° and the switch voltage of embossed bottom area (thinner layer) is about 1.5 V, approximately 1.5 V smaller than the coercive voltage of embossed top area (thicker layer). Rapid saturations are found at around 3 and 4 V, respectively. This provides convincing evidence of excellent ferroelectricity property of the embossed regions and demonstrates that two distinct coercive voltages can be generated by a one-step embossing process on ferroelectric thin films.

**Figure 3:**
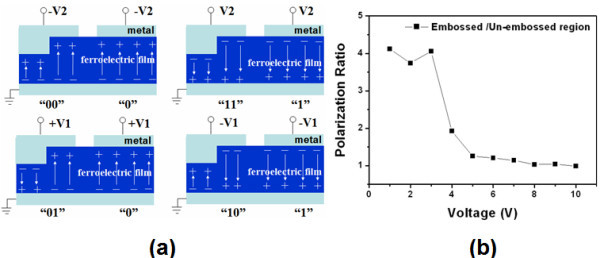
**Sketch map of multi-bit storage operation and remnant polarization comparison between an embossed and an un-embossed region**. **(a) **Schematically illustration of multi-bit storage operation for embossed regions on a PZT film. **(b) **Remnant polarization ratio of the embossed and the un-embossed regions in a PZT film in the voltage range from 1 to 10 V.

We propose a method for operation of multi-bit storage in embossed regions, which is schematically illustrated in Figure 3a. For instance, after a minus bias -V2, which is larger than the coercive voltage of the thicker film, is added both on the embossed and un-embossed regions, the polarization of the ferroelectric film under electrode pads could be wholly switched upward. Then with a plus voltage V1 applied on, which is smaller than the coercive voltage of the thicker film and larger than the coercive voltage of the thinner film, the polarization of the embossed bottom area can be switched downward. While for the embossed top area and un-embossed region, the polarization still keeps upward. In this way, an additional storage state for embossed region is apparently achieved. Indeed, Figure 3b plots the ratio of the remnant polarization of the embossed region to that of the un-embossed region on the same PZT film under voltages from 1 to 10 V. In the range of small voltages from 1 to 3 V, the polarization of the embossed area is about four times as large as that of the un-embossed region, and, with the voltage increasing to 5 V, the ratio decreases close to 1. This is attributed to the fact that the thinner layer in the embossed area could be more switched than the un-embossed region (coercive voltage is approx. 3 V) under low voltages such as 1 to 3 V. With the voltage rising to 5 V and above, both the embossed region and un-embossed region could be almost switched, and thus the remnant polarization for both regions with the same electrode pad areas (100 × 100 μm square) approaches a similar value.

**Figure 4:**
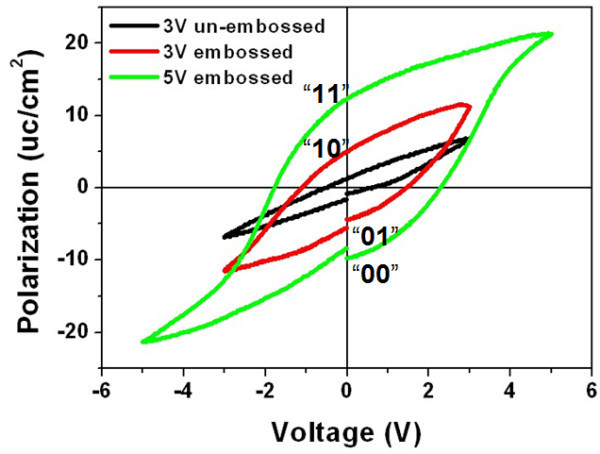
**Hysteresis loops of the embossed region in the voltages of 3 and 5 V, respectively**. The same loops obtained from the un-embossed region of the same PZT thin film is obtained under 3 V for comparison.

Figure 4 shows the hysteresis loops of the embossed region under external voltages of 5 and 3 V, respectively, as well as that of the un-embossed region on the same PZT thin film under 3 V for comparison. All of the hysteresis loops under varies voltages were measured from a pre-polarized state. The remnant polarization under 3 V for embossed region is 5.04 μc/cm^2^, while for un-embossed region is only 1.24 μc/cm^2^. This suggests two remnant polarization states can be created for '10' and '01' states by switching the polarization of thinner layer at a lower voltage. Correspondingly, when the polarization of whole layer was switched at a higher voltage, another two storage states '11' and '00' are obtained. As illustrated schematically in Figure 4, four storage states can be achieved in the embossed region.

**Figure 5:**
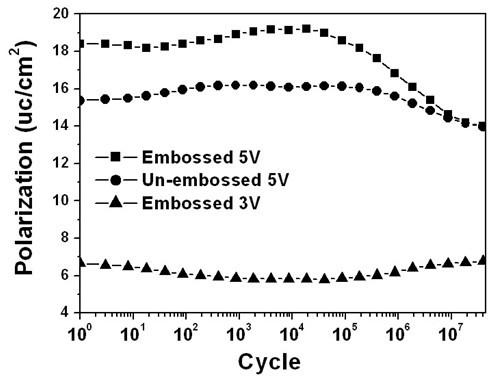
**Fatigue measurements from both embossed and un-embossed areas**. Change in switchable polarization as a function of the number of switching cycles for both embossed region with the biases of 5 and 3 V, respectively. Also shown in the figure is the switching behavior from the un-embossed region on the same PZT thin film under 5 V.

**Figure 6:**
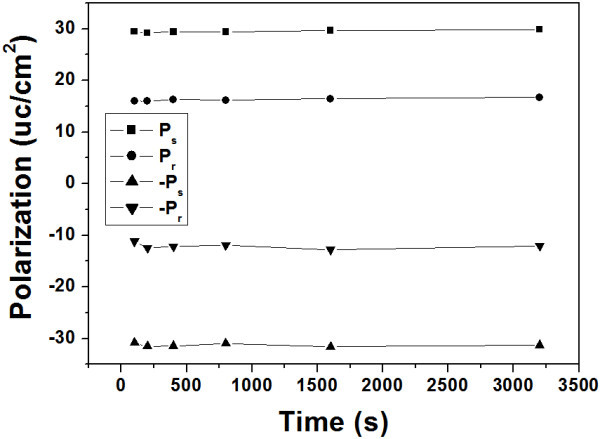
**Time dependence of the polarization on an embossed region from PUND measurements at room temperature**.

Fatigue measurements were done at room temperature using a Radiant Technologies Precision Tester. Both embossed and un-embossed regions were subjected to bipolar square wave voltage cycling with a width of 0.5 μs and period of 1 μs. Figure 5 displays the change in switchable polarization as a function of the number of switching cycles for both embossed and un-embossed regions on the same PZT thin film. It can be seen that un-embossed region is nearly fatigue free through 10^7 ^cycles under a voltage of 5 V, while the switchable polarization undergoes a slow decay starting at around 10^6 ^switching cycles for the embossed region under the same voltage. This could be attributed to the fact that the polarization of the thinner region in the embossed area was much more switched than the un-embossed region (coercive voltage is approx. 3 V) under the applied voltage, and thus underwent an earlier decay. Indeed, the switchable polarization of the embossed region reached as many as 10^7 ^switching cycles with no noticeable decay under a smaller voltage of 3 V. Further study on the fatigue [17-21] of the patterned ferroelectric film capacitor structure is underway. The retention characteristics of an embossed region have been obtained by a PUND method with the pulse width and amplitude of 1 ms and ± 8 V, respectively. Figure 6 shows the time dependence of the polarization, where *P*_s _is the switched polarization between the two opposite pulses and *P*_r _is the remnant polarization. As it can be seen that there is no polarization descending trend observed even after retention time of 3200 s, clearly confirming the excellent polarization phase maintained after the removal of external fields for a long time.

## Conclusions

In summary, we have successfully demonstrated a new method to fabricate multi-bit memory devices by embossing on thin PZT films at room temperature to form a stagger structure. More than one bit of data is obtained from a single ferroelectric memory cell with such a stagger configuration. Our process has the advantages of high throughput and low cost with a prospect for fabricating multi-bit memory devices by the developed embossing technique.

## Experiment details

Ferroelectric PZT thin films were prepared on Pt/Ti/SiO_2_/Si substrates by the sol-gel method. The raw materials were lead acetate trihydrate [Pb(OCOCH_3_)2.3H_2_O, 99.5%], zirconium tetra *n*-propoxide (Zr(OC_3_H_8_)_4_, 70%), titanium (IV) butoxide (Ti(OC_4_H_9_)_4_, 98%) as precursor material and Methanol/Acetic acid mixed solvent as a solvent. Figure 1a illustrates the embossing process of the PZT film. After spin-on, the precursor film was first baked on hotplate at 60°C in air for 5 min. Then, an embossing process was carried out at room temperature under a pressure of 9 Mpa for 15 min using a silicon template, which has a grating 500 nm lines/spaces. The template used was first coated with an anti-stick layer on its surface in order to reduce its adhesion to the embossed gels and make it easier in later de-mold procedure. After embossing, the gel layers were first pyrolyzed in air on a hotplate at 350°C for 5 min and then crystallized by conventional thermal annealing in air at 650°C for 15 min.

The PFM measurements of ferroelectric characteristics of structured PZT films were carried out at room temperature by a commercial multimode AFM with a Pt coated cantilever (force constant 0.03 to 0.2 N/m, and resonant frequency 14 to 28 kHz). An ac voltage of 1 V and frequency about 200 kHz was applied to measure the in-field nanoscale hysteresis loops.

Further electrical characterization of the embossed and un-embossed PZT ferroelectric films were performed using a Radiant Technologies Precision Material Analyzer with a triangular wave form at 1 kHz after forming Au/Cr electrode pads (100 × 100 μm square) on the films (Figure 3a).

## Abbreviations

AFM: atomic force microscopy; FeFETs: ferroelectric field effect transistors; FeRAMs: ferroelectric random access memories; OPP: out-of-plane piezoresponse; PFM: piezoresponse force microscopy.

## Competing interests

The authors declare that they have no competing interests.

## Authors' contributions

ZKS, ZHC, and QL designed and carried out the experiments. YFC and RL supervised the work. ZJQ, AQJ, and XPQ participated in the discussion. ZKS, YFC, and RL wrote the manuscript. All authors read and approved the final manuscript.

## Supplementary Material

Additional file 1 **XRD spectrum from an embossed region**. XRD spectrum from an embossed region suggests the embossed PZT film grown with the preferable [111] orientation ([100], [110] [200] and [211] peaks are much weaker). Click here for file

Additional file 2 **Raman spectrum taken from an embossed region**. The prominently intense, low-frequency modes at 141 and 199 cm^-1 ^relate to A_1_(1TO) and E(2TO)_T _mode, respectively. The peak at 504 and 602 cm^-1 ^correspond to E(3TO)_T _and A_1_(3TO) mode. These four modes are relating to the tetragonal structure of the embossed PZT film rather than the trigonal structure. Click here for file
