# Supplementary figures and images for: Nano-embossing technology on ferroelectric thin film Pb(Zr0.3,Ti0.7)O3 for multi-bit storage application
Source: Nanoscale Res Lett. 2011 Jul 27;6(1):474. doi: 10.1186/1556-276X-6-474 (PMC3211987; doi:10.1186/1556-276X-6-474)

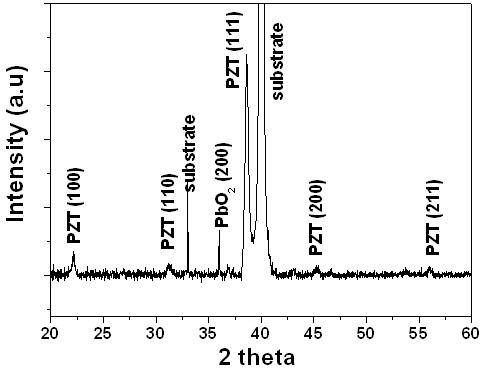

Supplement: Additional file 1 — XRD spectrum from an embossed region. XRD spectrum from an embossed region suggests the embossed PZT film grown with the preferable [111] orientation ([100], [110] [200] and [211] peaks are much weaker). [file 1556-276X-6-474-S1.BMP]

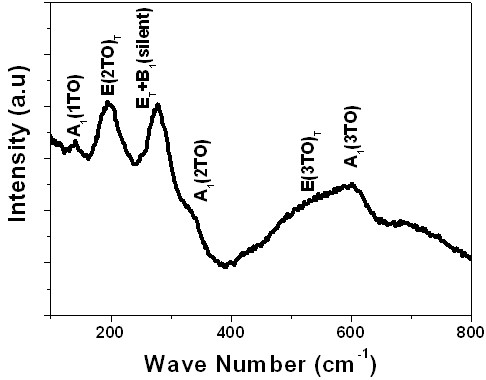

Supplement: Additional file 2 — Raman spectrum taken from an embossed region. The prominently intense, low-frequency modes at 141 and 199 cm-1 relate to A1(1TO) and E(2TO)T mode, respectively. The peak at 504 and 602 cm-1 correspond to E(3TO)T and A1(3TO) mode. These four modes are relating to the tetragonal structure of the embossed PZT film rather than the trigonal structure. [file 1556-276X-6-474-S2.BMP]
